# Factors associated with burnout among Chinese hospital doctors: a cross-sectional study

**DOI:** 10.1186/1471-2458-13-786

**Published:** 2013-08-29

**Authors:** Hui Wu, Li Liu, Yang Wang, Fei Gao, Xue Zhao, Lie Wang

**Affiliations:** 1 Department of Social Medicine, School of Public Health, China Medical University, No. 92 Beier Road, Heping District, Shenyang, Liaoning 110001, People’s Republic of China

**Keywords:** Burnout, Doctors, Maslach Burnout Inventory-General Survey, Occupational stress

## Abstract

**Background:**

Burnout has been a major concern in the field of occupational health. However, there is a paucity of research exploring the factors related to burnout among Chinese doctors. Investigation of these factors is important to improve the health of doctors and the quality of healthcare services in China.

**Methods:**

The study population consisted of 1,618 registered hospital doctors from Liaoning province of China. Burnout was measured using the Chinese version of the Maslach Burnout Inventory-General Survey. Occupational stress was measured using the Chinese versions of the Job Content Questionnaire and the Effort-Reward Imbalance Questionnaire. Data were collected on the respondents’ demographic characteristics and work situations. Of the doctors solicited for enrollment, 1,202 returned the completed questionnaire (555 men, 647 women), giving a response rate of 74.3%. A general linear regression model was applied to analyze the factors associated with burnout.

**Results:**

The burnout mean scores were 11.46 (7.51) for emotional exhaustion, 6.93 (5.15) for cynicism, and 24.07 (9.50) for professional efficacy. In descending order of standardized estimates, variables that predicted a high level of emotional exhaustion included: high extrinsic effort, dissatisfaction with doctor-patient relationship, high overcommitment, working >40 h per week, low reward, and high psychological job demands. Variables that predicted a high level of cynicism included: high extrinsic effort, low reward, dissatisfaction with doctor-patient relationship, high overcommitment, low decision authority, low supervisor support, and low skill discretion. Variables that predicted a low perceived professional efficacy included: high psychological job demands, low coworker support, high extrinsic effort, low decision authority, low reward, and dissatisfaction with doctor-patient relationship.

**Conclusions:**

These findings suggest that occupational stress is strongly related to burnout among hospital doctors in China. Strategies that aim to improve work situations and decrease occupational stress are necessary to reduce burnout, including health education, health promotion, and occupational training programs.

## Background

Burnout is defined as “a syndrome of exhaustion, cynicism and low professional efficacy” [1]. Researchers and administrators are increasingly recognizing the problem of burnout, which has been a major concern internationally in the field of occupational health [2-5]. Studies have found that burnout may lead to increased rates of absenteeism and turnover, and decreased job performance, commitment, career satisfaction, and productivity [6-10].

As human service professionals, doctors are exposed to a range of factors such as work overload, time pressures, role conflicts, and effort–reward imbalances. Further, the relationship between doctors and patients often involves high interpersonal and/or emotional demands; this can also elevate the risk of burnout [2,10-13]. Doctors who experience burnout have a high incidence of depressive symptoms and suicide attempts, and are often unable to meet the demands of patients [14-16].

China is currently undergoing a major healthcare system reform; the focus is transforming from disease to health, and from sustaining life to quality of life. Moreover, the huge population base and increasing health consciousness over recent years have led to an overload of patients for Chinese doctors. In China, the ratio of doctors to the general population is 1:735, which is considerably lower than that of Western countries (1:280–1:640) [17]. Chinese doctors frequently experience work overload and extra shifts and, at times, disputes occur because of patient dissatisfaction with care [13,18]. Doctors are more likely to experience time pressures and energy deficiencies, which seem to aggravate the burnout of Chinese doctors [19].

Results from previous studies suggest that burnout among doctors is the result of exposure to a combination of factors related to their personal and working environments. These factors are believed to be demographic (age, marital status, and education) [12,20] and work related (work time, work shift, and role boundaries) [20,21]. Occupational stress has been identified as the major factor associated with burnout [11,22,23]. Studies of occupational stress have considered various theoretical approaches, including the Job Demand–Control (JDC) model [24] and the Effort–Reward Imbalance (ERI) model [25,26]. The JDC model postulates that the source of job stress comes from two basic characteristics of the job itself: psychological job demands and job control (known in the model as ‘decision latitude’), and social interactions (‘social support’ as per the model). Psychological strain is a consequence of the combined effects from the job’s demands and control on an individual, while low social support at work further aggravates occupational stress. The ERI model emphasizes the non-reciprocal social exchange between costs and gains at work and overcommitment, which can cause a state of emotional distress and lead to adverse health outcomes. The two models complement each other in that the JDC model focuses on the task characteristics and social aspects of the workplace, while the ERI model relates to the stressful experiences and personal cognitive patterns of dealing with work. However, the association between occupational stress and burnout has received less attention, especially in relation to the combined effects of the JDC model and the ERI model. Therefore, factors from both models were included in this study.

Burnout among hospital doctors has been widely studied in Western countries. Some research has been undertaken in China to explore the factors that contribute to the burnout of doctors [13,18], where the health system and culture may result in a different experience for doctors. For example, because of patient overload in most Chinese hospitals, doctors work 40 h or more each week. There are no on-call doctors for sick leave or holidays [27]. In addition, the characteristics of Chinese culture are also different when compared with Western countries. There are hierarchical social rules between parents and children, and older and younger people; these rules are strictly observed in all aspects of the social lives of Chinese people [28].

During the recent health sector reforms, doctors have not only been overloaded, but their average income level has been relatively low. As a consequence, ERI could be a factor associated with burnout. In addition, doctors exhausted with their heavy patient load might have found it difficult to give patients the care they deserve. We hypothesize that the doctor-patient relationship is an important factor related to burnout. Taking into consideration the conclusions from previous studies and the different health system and culture in China, in the current study, the following factors were investigated for their association with burnout, with the aim of reducing burnout among Chinese doctors: demographics, work situations (including the doctor-patient relationship), and occupational stress (including ERI).

## Methods

### Subjects and procedures

China Medical University is the leading medical institution in northeast China. It has seven teaching hospitals dispersed throughout Liaoning province with a total of 1,629 registered doctors. A cross-sectional survey was conducted during May and June 2010. All seven teaching hospitals were approached; the principal investigators met with hospital administrators to share the study questionnaire. The administrators assisted in contacting the eligible doctors who had agreed to participate. After a verbal briefing from the principal investigators regarding the study goals and the information being obtained by the questionnaire, the doctors could choose whether or not to complete the study. Informed consent was obtained from participants; individualized data were kept confidential and was only used for the study’s analysis. Eleven refused to participate (five men). The questionnaires were eventually distributed to the 1,618 registered doctors who agreed to participate. Because of limited available free time during work hours, the questionnaires were self-administered by the doctors and returned to the administrators. The principal investigators collected the completed questionnaires at an appointment with the administrators 1 week later. A total of 1,202 completed questionnaires were received (555 men and 647 women) yielding a response rate of 74.3%.

The study was approved by the Committee on Human Experimentation at China Medical University (Senyang, Liaoning, China).

### Measurement of burnout

Burnout was measured using the Chinese version of the Maslach Burnout Inventory-General Survey (MBI-GS) [1]. The MBI-GS was translated and back-translated to ensure it reflected the original message, and revised to make the items culturally and linguistically applicable to China [27]. A Chinese version of the MBI-GS has previously been used with Chinese nurses and doctors [13,18,29-31]. The survey includes three subscales: emotional exhaustion (EE; five items), cynicism (CY; four items), and professional efficacy (PE; seven items). Each item has seven possible answers: never, rarely (several times per year), sometimes (once a month), often (several times per month), frequently (once a week), always (several times per week), and every day. These responses were scored from 0 to 6. The possible minimum and maximum scores were from 0 to 30 for EE, from 0 to 24 for CY, and from 0 to 42 for PE. Emotional exhaustion was defined as feeling emotionally overwhelmed and exhausted by work; CY was defined as doubting the value of one's work or its contribution to anything. Reduced PE described a feeling of reduced competence and a lack of success/achievement in one’s work with people. The cut-off points of the MBI-GS in the Chinese version were: EE – low < 9, average 9–13, high >13; CY – low < 3, average 3–9, high > 9; PE – low > 30, average 30–18, high < 18. Individuals with high EE and CY scores combined with a low PE score were identified as having a high degree of burnout [27]. In this study, Cronbach alpha coefficients for EE, CY, and PE were 0.95, 0.92, and 0.88, respectively.

### Demographics

Demographics collected from each respondent included age, gender, education, and marital status. Age was classified as ≤ 30, 31–40, 41–50, or ≥ 51 years. The education classification was based on the doctors’ educational experiences before they started working. In China, health workers who graduate from junior college are able to obtain a doctor’s license after becoming a licensed assistant doctor and gaining experience with medical practice. As such, education was grouped into three different categories: junior college, undergraduate, and graduate. Marital status was categorized as married or single. In this study, only 13 participants (1.08%) belonged to the ‘widowed/divorced” group; this group was combined with the ‘single’ group.

### Work situations

Work situations were defined as job rank, weekly work time and doctor-patient relationship. Job rank was categorized as head doctor or staff doctor by asking the question, “Are you a head doctor or a staff doctor?” Weekly work time was divided into ≤ 40 h or > 40 h, according to the current work arrangement of 8 h per day. Doctor-patient relationship was assessed by the question, “How often have you been dissatisfied with the doctor-patient relationship at work?” with five possible answers: never, rarely, sometimes, frequently, and always; the responses were further dichotomized into ‘general dissatisfaction’ (never/rarely/sometimes) or ‘serious dissatisfaction’ (frequently/always) [32,33].

### Occupational stress

Occupational stress was measured using the Chinese version of the Job Content Questionnaire (JCQ), based on the JDC model, and the Effort–Reward Imbalance (ERI) Questionnaire, based on the ERI model. The JCQ and ERI have been reported to have good reliability and validity in the Chinese population [34,35].

The 22-item JCQ has three scales: decision latitude, psychological job demands, and social support. The decision latitude scale is the sum of two subscales: skill discretion (measured by six items) and decision authority (measured by three items). The psychological job demands scale is measured by five items. The social support scale is the sum of two subscales: coworker support (measured by four items) and supervisor support (measured by four items). Responses to all the items were scored from 1, for complete disagreement, to 4 for complete agreement. The Cronbach alpha coefficients for decision latitude, psychological job demands, and social support were 0.83, 0.88, and 0.91, respectively.

The ERI contains 23 items that measure extrinsic effort (six items), reward (11 items), and overcommitment (six items). Each response to extrinsic effort and reward was scored from 1 to 5; higher total scores indicated higher demands of efforts and higher rewards. Responses to overcommitment were scored from 1, for complete disagreement, to 4 for complete agreement. Higher scores suggest higher demands characterized by excessive work-related commitments. The Cronbach alpha coefficients for extrinsic effort, reward, and overcommitment were 0.90, 0.81, and 0.86, respectively.

### Data analysis

Data were analyzed using Statistical Analysis System for Windows, version 8.2 (SAS Institute Inc., Cary, NC, USA). Before the data analyses were conducted, the normal distribution of the variables was tested. The P-P-plot analyses and K-S tests of normal distribution indicated that the variables fulfilled the postulation of normal distribution. Correlations between independent variables were tested by Kappa test for the categorical variables and by Pearson correlation for the continuous variables. If the Kappa value and correlation coefficient between independent variables was more than 0.5, these variables were regarded as having agreement and were adjusted using multivariate analysis. In this study, no agreement was found. Comparisons of MBI-GS scores for demographics and work situations were tested by the Student t-test, ANOVA, and post-hoc test. Correlations between MBI-GS scores and occupational stress were examined by Pearson correlation. Factors associated with MBI-GS scores were identified by a general linear model with adjustment for age and gender. The following variables were entered into a general linear analysis: demographics (age, education, and marital status), work situations (job rank, weekly work time, and doctor-patient relationship), and occupational stress (JDC and ERI). The effect of different variables on the variance in MBI-GS subscale scores was tested by hierarchical linear regression analysis. In step 1, demographics were added. In step 2, work situations were added. In step 3, occupational stress was added. Statistical significance was defined as *p* < 0.05.

## Results

The average age of respondents was 38.67 (8.78) years; 38.86 (9.30) years for men and 38.51 (8.31) years for women. The burnout mean scores were 11.46 (7.51) for EE, 6.93 (5.15) for CY, and 24.07 (9.50) for PE. Respondent distribution by demographics and work situations is shown in Table 1. This shows that 46.2% were male and 53.8% were female, 94.3% of respondents had at least attained an undergraduate qualification, and 80.9% were married. Among all the respondents, 17.5% were head doctors, 61.9% worked > 40 h per week, and 47.2% reported serious dissatisfaction with their doctor-patient relationships. The prevalence of a high degree of burnout was 12.1% in this study.

**Table 1:** Respondent distribution by demographics and work situations

**Variables**	**N (%)**
Age (years)	
≤ 30	295 (24.5)
31-40	406 (33.8)
41-50	375 (31.2)
≥ 51	126 (10.5)
Gender	
male	555(46.2)
female	647(53.8)
Education	
Junior college	68 (5.7)
Undergraduate	809 (67.3)
Graduate	325 (27.0)
Marital status	
Married	972 (80.9)
Single	230 (19.1)
Job rank	
Head doctor	210 (17.5)
Staff doctor	992 (82.5)
Weekly work time (hours)	
≤ 40	458 (38.1)
> 40	744 (61.9)
Doctor-patient relationship	
General dissatisfaction	635 (52.8)
Serious dissatisfaction	567 (47.2)

Univariate analysis of MBI-GS scores in relation to demographics and work situation variables are shown in Table 2. There was no significant association of burnout scores based on gender or marital status. Age, education, job rank, weekly work time and the doctor-patient relationship were significantly related to EE and CY scores (*p* < 0.05). For age, EE was significantly lower among those aged ≥ 51 years when compared with other groups; CY was significantly higher among those aged ≤ 30 years than those aged 41–50 and ≥ 51 years, and CY was significantly higher among those aged 31–40 years than those aged 41–50 and ≥ 51 years. For education, EE was significantly higher among graduate than junior college and undergraduate groups; EE was significantly higher among undergraduate group compared with junior college group. Further, the CY scores were significantly lower among junior college group than undergraduate and graduate groups. Weekly work time and doctor-patient relationship were significantly related to PE scores (*p* < 0.05).`

**Table 2:** Univariate analysis of MBI-GS scores in relation to demographics and work situations

**Variables**	**MBI scores (Mean ± SD )**		
	**EE**	**CY**	**PE**
Age (years)			
≤ 30	12.40 ± 7.75*	7.67 ± 5.71*	23.35 ± 8.77
31-40	11.88 ± 7.16*	7.46 ± 4.97*	23.61 ± 9.87
41-50	11.00 ± 7.51*	6.28 ± 4.95	24.19 ± 10.28
≥ 51	9.26 ± 7.64	5.45 ± 4.55	25.09 ± 9.32
Gender			
male	11.61 ± 7.86	7.43 ± 5.42	23.57 ± 9.39
female	11.32 ± 7.21	7.10 ± 4.88	24.49 ± 9.58
Education			
Junior college	8.93 ± 6.91	5.26 ± 4.31	24.18 ± 9.39
Undergraduate	12.26 ± 7.47*	7.53 ± 5.39*	24.06 ± 9.21
Graduate	13.96 ± 7.98*	7.87 ± 4.70*	23.79 ± 9.86
Marital status			
Married	11.10 ± 7.03	6.73 ± 5.16	24.26 ± 9.45
Single	11.54 ± 7.62	6.98 ± 5.14	23.24 ± 9.68
Job rank			
Head doctor	9.29 ± 7.19	5.42 ± 4.62	24.19 ± 9.60
Staff doctor	11.92 ± 7.50**	7.25 ± 5.21**	24.04 ± 9.48
Weekly work time (hours)			
≤ 40	9.70 ± 6.75	5.77 ± 4.61	24.19 ± 9.45
> 40	11.94 ± 7.64**	7.25 ± 5.26**	23.63 ± 9.70**
Doctor-patient relationship			
General dissatisfaction	8.24 ± 5.84	4.57 ± 3.44	24.50 ± 9.92
Serious dissatisfaction	15.06 ± 7.54**	9.58 ± 5.87**	23.58 ± 8.99*

*p < 0.05, **p < 0.01.

*MBI-GS*, Maslach Burnout Inventory-General Survey.

*EE*, Emotional exhaustion; *CY*, Cynicism; *PE*, Professional efficacy.

Correlations between MBI-GS scores and occupational stress are detailed in Table 3. The following JCQ variables showed negative correlations with both EE and CY, and positive correlations with PE (*p* < 0.01): skill discretion, decision authority, supervisor support, and coworker support. Psychological job demands had a positive correlation with EE and CY, and a negative correlation with PE (*p* < 0.01). For the ERI variables, both extrinsic effort and overcommitment showed positive correlations with EE and CY, and negative correlations with PE (*p* < 0.01). Reward had a negative correlation with EE and CY, and a positive correlation with PE (*p* < 0.01).

**Table 3:** Correlations between MBI-GS scores and occupational stress

**Variables**	**MBI-GS scores ( *r *)**		
	**EE**	**CY**	**PE**
JCQ			
Skill discretion	−0.11**	−0.16**	0.07**
Decision authority	−0.19**	−0.24**	0.09**
Psychological job demands	0.39**	0.21**	−0.13**
Supervisor support	−0.22**	−0.24**	0.08**
Coworker support	−0.10**	−0.15**	0.11**
ERI			
Extrinsic effort	0.52**	0.46**	−0.04
Reward	−0.43**	−0.43**	0.08**
Overcommitment	0.49**	0.34**	−0.10**

**p < 0.01.

*MBI-GS*, Maslach Burnout Inventory-General Survey.

*EE*, Emotional exhaustion; *CY*, Cynicism; *PE*, Professional efficacy.

A general linear model analysis of the factors associated with MBI-GS scores is shown in Table 4. After controlling for age and gender, variables that predicted a high level of EE (in descending order of standardized estimate) included: high extrinsic effort, dissatisfaction with doctor-patient relationship, high overcommitment, working > 40 h per week, low reward, and high psychological job demands (*F*_16,1185_ = 154.82, *p* = 0.00). Variables that predicted a high level of CY (in descending order of standardized estimate) included: high extrinsic effort, low reward, dissatisfaction with doctor-patient relationship, high overcommitment, low decision authority, low supervisor support, and low skill discretion (*F*_16,1185_ = 64.53, *p* = 0.00). Variables that predicted a low level of PE (in descending order of standardized estimate) included: high psychological job demands, low coworker support, high extrinsic effort, low decision authority, low reward, and dissatisfaction with doctor-patient relationship (*F*_16,1185_ = 9.92, *p* = 0.00).

**Table 4:** General linear model analysis of the factors associated with MBI-GS scores

**Variables**	**MBI-GS scores**					
	**EE**		**CY**		**Low PE**	
	**B**	**Beta**	**B**	**Beta**	**B**	**Beta**
Weekly work time						
(≤ 40 hours vs. > 40 hours)	−1.754**	−0.096**				
Doctor-patient relationship						
(Serious dissatisfaction vs. General dissatisfaction)	2.535**	0.169**	2.273**	0.159**	1.287*	0.068*
Skill discretion			−0.109*	−0.069*		
Decision authority			−0.110**	−0.091**	−0.137**	−0.085**
Psychological job demands	0.068*	0.065*			0.230**	0.118**
Supervisor support			−0.282**	−0.090**		
Coworker support					−0.592**	−0.107**
Extrinsic effort	0.607**	0.401**	0.236**	0.235**	0.368**	0.097**
Reward	−0.144**	−0.074**	−0.230**	−0.199**	−0.103*	−0.077*
Overcommitment	0.464**	0.154**	0.287**	0.101**		

*p < 0.05, ** *p* < 0.01. Age and gender were controlled in the model.

*MBI-GS*, Maslach Burnout Inventory-General Survey.

*EE*, Emotional exhaustion; *CY*, Cynicism; *PE*, Professional efficacy.

B, Unstandardized coefficients.

Beta, Standardized coefficients.

The effect of different variables on the variance in MBI-GS subscale scores is detailed in Table 5. Demographic variables explained 1% of variance in EE, CY, and PE. Work situations explained 12% of variance in EE, 10% of variance in CY, and 6% of variance in PE. Occupational stress was responsible for 32%, 22%, and 7% of variance in EE, CY, and PE, respectively.

**Table 5:** The effect of different variables on the variance in MBI-GS subscale scores

**Variables**		**EE**	**CY**	**low PE**
Demographics	R^2^	0.01	0.01	0.01
	∆R^2^	0.01	0.01	0.01
	F	3.92**	4.25**	2.86
Work situations	R^2^	0.13	0.11	0.07
	∆R^2^	0.12	0.10	0.06
	F	23.78**	24.15**	6.16**
Occupational stress	R^2^	0.45	0.33	0.14
	∆R^2^	0.32	0.22	0.07
	F	63.64**	37.59**	10.25**

**P < 0.01.

*MBI-GS*, Maslach Burnout Inventory-General Survey.

*EE*, Emotional exhaustion; *CY*, Cynicism; *PE*, Professional efficacy.

∆R^2^, R^2^ increase.

In step 1, demographics were added.

In step 2, work situations were added.

In step 3, occupational stress was added.

## Discussion

This study explored the factors associated with burnout among hospital doctors in China. Our study subjects were drawn from seven teaching hospitals of the China Medical University, Liaoning province, which has a proportion of doctors in the health care work force (34.5%) comparable with the national proportion of doctors (35.4%) with the same average income level as the average national level in China [36]. The burnout mean scores were 11.46 (7.51) for EE, 6.93 (5.15) for CY, and 24.07 (9.50) for PE. The levels of EE and CY were higher than the levels of other occupations that have been previously reported in China, and the level of PE was lower than other occupations [30,37]. The prevalence of a high degree of burnout in this study was 12.1%, which is higher than rates from other countries [38,39]. This finding should encourage hospital administrators to be aware about the risk of burnout. Efforts should be made to prevent and reduce burnout among Chinese doctors.

In this study, work situations explained 12% of variance in EE, 10% of variance in CY, and 6% of variance in PE. Among the factors related to work situations, weekly work time and the doctor-patient relationship are two factors that have been shown to be related to burnout, both in this and previous studies [20,21]. Doctors in the current study who worked more than 40 h per week and reported dissatisfaction with doctor-patient relationship had significantly higher EE and CY scores and lower PE scores than respondents who worked fewer hours. Interestingly, 47.2% of doctors in this study reported serious dissatisfaction with their relationship with patients; this was one of the most salient factors related to high EE and CY, and low PE scores. One possible explanation is that the Chinese healthcare service has been rapidly changing over recent years, and the traditional disease-centered care model is gradually being replaced by a patient-centered approach. Thus, many doctors’ responsibilities have increased, as have their patients’ demands [40]. In addition, the huge population base in China and the corresponding increase in patient numbers have created a very large work burden for doctors. As a result, doctors are working longer hours and have less time for communicating with patients, and medical disputes often occur owing to patient dissatisfaction with care [18,41]. These combined effects have created an increasing conflict between patients and doctors and strained the doctor-patient relationship [19,33]. As a consequence, organizational interventions such as decreasing long working hours and improving the doctor-patient relationship should be considered during the current reformation of care models [19].

Occupational stress measured by the JCQ and ERI was found to be the major factor associated with burnout [4,42]. Our results also indicate that all eight subscales in the JCQ and ERI had high correlations with the EE and CY burnout subscales, some of which were also associated with low PE; this is in agreement with studies previously cited in this manuscript. In our study, occupational stress explained 32% of variance in EE, 22% of variance in CY, and 7% of variance in low PE. High extrinsic effort was the most powerful indicator of EE and CY; high psychological job demands were the most robust indicator of low PE. A possible explanation for this result is that patient overload of doctors is especially serious in China because of the country’s population base of 1.3 billion people. Overload of patients, together with the care model transition, has resulted in a high workload for doctors [41]. Many doctors have increased their extrinsic effort and psychological job demands to meet these changes. When the job demands exceed the capability of doctors, and their efforts exceed reward, the impact on their physical and mental health can lead to burnout [13,19].

Previous studies have shown that relationships with superiors and colleagues are factors that influence burnout [22,23,43]. Poor relationships with superiors and colleagues may lead to feelings of exclusion and loneliness among doctors. Burnout may occur because there are no avenues for sharing their sense of frustration, or for receiving positive feedback from their peers. Conversely, good relationships with superiors and colleagues are protective factors [44]. Our study reported similar results; supervisor support and colleagues support has an important influence on burnout. The findings in this study contribute to our understanding that high levels of supervisor and colleague support has a positive effect on combating burnout among doctors. Reducing occupational stress and strengthening relationships with superiors and colleagues may decrease burnout and improve doctors’ quality of life, as observed in other studies [19,41].

### Limitations

Despite these merits, there are several limitations in the present study. First, participants in this study were limited to teaching hospitals of the China Medical University. Although the hospitals are dispersed across a range of city sizes, accurate representation of our study population may not be complete. Second, this study used a cross-sectional design, making it difficult to study causal relationships. Third, we have no information about the non-responders’ characteristics, which may have introduced response bias. Fourth, this study was a preliminary investigation and as such did not address all medical specialties and the wide range of factors (e.g., sleep problems, and physical, cognitive, and emotional demands). Fifth, the doctor-patient relationship issue was measured using a single question; a more substantial questionnaire should have been used to address the doctor-patient relationship dynamic. These factors should be considered in further studies.

## Conclusions

Occupational stress was found to be the most robust indicator of burnout among Chinese hospital doctors. Burnout was predicted by such factors as strong extrinsic effort, the most powerful indicator of EE and CY, and strong psychological job demands, the most robust indicator of low PE. Our results highlight the importance of addressing extrinsic efforts to prevent EE and CY, as well as addressing psychological job demands to prevent low PE. Our findings underscore the need for medical leaders to be aware of the risk of burnout. Efforts should be made to develop strategies to improve working conditions and reduce occupational stress, thereby mitigating the risk of burnout in doctors.

## Competing interests

The authors declare that they have no competing interests.

## Authors’ contributions

HW designed the research, carried out data analysis, and wrote the paper. LW provided guidance in study design, organized the investigation, and is the corresponding author. LL, FG, and XZ provided help with the data collection, analysis, interpretation, and write-up. YW provided help with the data collection and interpretation. All authors read and approved the final manuscript.

## Pre-publication history

The pre-publication history for this paper can be accessed here:

http://www.biomedcentral.com/1471-2458/13/786/prepub
